# A Knowledge Graph of Combined Drug Therapies Using Semantic Predications From Biomedical Literature: Algorithm Development

**DOI:** 10.2196/18323

**Published:** 2020-04-28

**Authors:** Jian Du, Xiaoying Li

**Affiliations:** 1 National Institute of Health Data Science Peking University Beijing China; 2 Institute of Medical Information Chinese Academy of Medical Sciences Beijing China

**Keywords:** combined drug therapy, knowledge graph, knowledge discovery, semantic predications

## Abstract

**Background:**

Combination therapy plays an important role in the effective treatment of malignant neoplasms and precision medicine. Numerous clinical studies have been carried out to investigate combination drug therapies. Automated knowledge discovery of these combinations and their graphic representation in knowledge graphs will enable pattern recognition and identification of drug combinations used to treat a specific type of cancer, improve drug efficacy and treatment of human disorders.

**Objective:**

This paper aims to develop an automated, visual approach to discover knowledge about combination therapies from biomedical literature, especially from those studies with high-level evidence such as clinical trial reports and clinical practice guidelines.

**Methods:**

Based on semantic predications, which consist of a triple structure of subject-predicate-object (SPO), we proposed an automated algorithm to discover knowledge of combination drug therapies using the following rules: 1) two or more semantic predications (S_1_-P-O and S_i_-P-O, i = 2, 3…) can be extracted from one conclusive claim (sentence) in the abstract of a given publication, and 2) these predications have an identical predicate (that closely relates to human disease treatment, eg, “treat”) and object (eg, disease name) but different subjects (eg, drug names). A customized knowledge graph organizes and visualizes these combinations, improving the traditional semantic triples. After automatic filtering of broad concepts such as “pharmacologic actions” and generic disease names, a set of combination drug therapies were identified and characterized through manual interpretation.

**Results:**

We retrieved 22,263 clinical trial reports and 31 clinical practice guidelines from PubMed abstracts by searching “antineoplastic agents” for drug restriction (published between Jan 2009 and Oct 2019). There were 15,603 conclusive claims locally parsed using the search terms “conclusion*” and “conclude*” ready for semantic predications extraction by SemRep, and 325 candidate groups of semantic predications about combined medications were automatically discovered within 316 conclusive claims. Based on manual analysis, we determined that 255/316 claims (78.46%) were accurately identified as describing combination therapies and adopted these to construct the customized knowledge graph. We also identified two categories (and 4 subcategories) to characterize the inaccurate results: limitations of SemRep and limitations of proposal. We further learned the predominant patterns of drug combinations based on mechanism of action for new combined medication studies and discovered 4 obvious markers (“combin*,” “coadministration,” “co-administered,” and “regimen”) to identify potential combination therapies to enable development of a machine learning algorithm.

**Conclusions:**

Semantic predications from conclusive claims in the biomedical literature can be used to support automated knowledge discovery and knowledge graph construction for combination therapies. A machine learning approach is warranted to take full advantage of the identified markers and other contextual features.

## Introduction

### Background

Combination drug therapy is a therapeutic intervention in which multiple drugs are administered, particularly in patients with malignant neoplasms [1,2]. Compared with single-agent therapy, the synergistic interaction of combined medications significantly improves drug efficacy, shortens disease course, delays or avoids drug resistance, and reduces both toxicity and other side effects without loss of efficacy. The combination of several existing drugs with compatible mechanisms of action has been reported as an alternative approach to advance the success of drug repositioning [3]. The characteristics of combination therapies make them a practical alternative to standard approaches, with the potential to save billions of dollars on research and development of new drugs, particularly in the absence of effective monotherapies for many types of cancer and other diseases (such as autoimmune and psychiatric conditions), and more than 6700 rare diseases for which no therapies are available [3].

In recent decades, massive efforts have been made to employ combined therapeutic agents to improve treatment of human disorders such as specific cancers [2,4], malignancies such as lymphocytic leukemia [1], and hypertension [5]. PubMed houses over 175,000 publications found by searching the MeSH (Medical Subject Headings) heading “Drug Therapy, Combination” (Jan 2009 to Oct 2019). We used innovative information retrieval and semantic web technologies to discover knowledge about therapeutic drug combinations, then presented the findings in a visually intuitive knowledge graph. The resulting knowledge graph will not only support machine-understandable information for curing disease and drug efficacy screening, but also provide insights to quickly develop new therapies for untreated diseases.

In this paper, we propose a systematic, automated approach to discover knowledge about combination drug therapies in the biomedical literature (especially clinical trial reports and clinical practice guidelines with high evidence levels), and integrate the findings into knowledge graphs with customized organization and visualization. This entails the following:

1. Propose an automated algorithm to discover knowledge about combination drug therapies based on semantic predications extracted from conclusive claims in biomedical literature
2. Customize a knowledge graph to emphasize the specified drugs being combined rather than traditional triples (eg, one drug TREATS one disease)
3. Retrieve published clinical trial reports and clinical practice guidelines for algorithm verification and validation, followed by manual identification of accurate knowledge about combination drug therapies, as well as interpretation of inaccurate findings
4. Characterize the major patterns of combinations according to mechanism of action for new combined medication studies and identify potential markers as key features for machine learning-based drug combination discovery.

In the following sections, we review related work on knowledge graphs and drug-disease knowledge discovery. We then present our methodology to develop an automated algorithm to discover knowledge about combination drug therapies. A large number of clinical trial reports and clinical practice guidelines were retrieved from PubMed for algorithm verification and validation, followed by manual biocuration to verify accurate results for knowledge graph construction and to interpret inaccurate results. In the discussion we characterize the main patterns of drug combinations according to their mechanisms of action to inform new combination studies and identify markers of potential combined drug therapies to inform machine learning–based algorithm development.

### Related Work

#### Knowledge Graph

A knowledge graph is a network-based representation of the semantic relationship between entities. Its principles have been developed by industry and academia, particularly by the semantic web community. In 1982, Hoede and Stokman used large graphs to represent knowledge extracted from medical and sociology texts [6], resulting in an expert system for quick searching and decision support for automated queries. In 2012, Google formally introduced their knowledge graph after compiling over 3.5 billion facts and relationships among 500 million objects, which is essentially a semantic enhancement of the search engine to help search real-world objects quickly and easily. At the end of 2016, Microsoft announced a large graph of concepts harnessed from billions of web pages and search logs for short text understanding, called the Concept Graph. Other frequently mentioned applications are Yahoo Spark, Facebook’s entity graph, Wikidata, Freebase, Baidu’s Knowledge Graph, and Sogou’s Knowledge Cube. Although these products differ in their architecture, operational purpose, and supported technologies, they constitute a family of knowledge graphs and together represent the precursor to a new generation of semantic search and knowledge discovery.

Many other studies on biomedical knowledge graphs have been performed since 2012, playing an indispensable role in biomedical knowledge services. Remarkable achievements encompass the organization of health information from heterogeneous textual [7], disease-symptom association learning from electronic medical records [8], presenting relationships between cells and cytokines [9], extraction of human disorder biomarkers [10], and predicting drug efficacy [11]. However, knowledge graphs have not yet been applied to organize and manage biomedical information related to combination drug therapies, especially when such knowledge comes from the direct empirical evidence of clinical research.

#### Biomedical Drug-Disease Knowledge Discovery

Studies on biomedical knowledge discovery mainly focus on the semantic relationships, associations, and interactions between biomedical entities such as diseases, drugs, signs or symptoms, target organ, genes, biomarkers, and targets. One of the most important tasks is to identify the exact relationship between a drug and disease, especially for “treatment.” Many information retrieval techniques and methods have been used to approach this problem based on predefined rules [12,13] or natural language processing [14-19] combined with machining learning [17-19]. Although predefined rules offer promising precision from biomedical texts, they are insufficient and perform poorly when parsing big data due to the noisy and variable syntactic structures within large-scale scientific texts. In comparison, natural language processing-based algorithms have generally been more successful and relatively flexible by virtue of features that parse context in literature.

Semantic Knowledge Representation, or SemRep, is a natural language processing tool based on the Unified Medical Language System (UMLS) [20]. This high-quality tool for extracted semantic predication has already been utilized for a broad range of applications such as the construction of a biomedical knowledge graph [21], identification of apparent contradictions [22], labeling for semantic relationships [23], and detection of drug-drug interactions [24] or drug-gene targets [25]. Here, we extend the application scope of SemRep by using semantic predications from conclusive sentences (eg, the conclusion section) of abstracts in biomedical literature, rather than the whole abstract, to automatically discover knowledge about combination drug therapies. The conclusion statement of a paper is the essential knowledge unit that synthesizes the knowledge content of an article and is validated by the experiment reported within the article.

## Methods

### Using Conclusive Sentences in the Abstract of a Publication as Knowledge Claims

There is a vast amount of published biomedical literature easily available in digital and printed format due to the rapid advance of information technology. For example, the cumulative citations of PubMed resources have exceeded 25 million, expanding with an annual growth of 0.9 million [26]. The huge amount of literature encourages the emergence of automated knowledge discovery, which could help scientists keep up with the latest scientific developments and academic achievements.

Scientific publications can be considered records of knowledge claims on a research question, supported by empirical evidence. These knowledge claims are often succinctly described in the abstract of a publication. The abstract is the most frequently accessed section of a publication and the only section used as source information in indexing databases such as PubMed. In this study, we parsed abstracts from PubMed for conclusive claims identified by the key words “conclusion*” and “conclude*” (Table 1) in order to discover knowledge about combination drug therapies.

### Semantic Predication Interpretation Using SemRep

SemRep is a well-developed semantic knowledge interpreter that retrieves semantic predications (in terms of subject-predicate-object) to extract information from biomedical texts. For example, for the first claim in Table 1, SemRep would interpret the 7 semantic predications shown in Table 2, and the predications with “INFER” in the predicate was inferred based on two existing predications.

As a natural language processing driven tool, SemRep takes full advantage of UMLS knowledge sources including the Metathesaurus and Semantic Network. Briefly, the subject and object of semantic predication returned by SemRep are the preferred names of biomedical concepts in the UMLS Metathesaurus, while the predicates were derived from semantic relationships in the UMLS Semantic Network. An evaluation based on sample data with semantic type “Chemicals and Drugs” has allowed SemRep to achieve a promising degree of precision (83%) [20], which will contribute to the development of algorithms for automated knowledge discovery for combination drug therapy.

**Table 1:** Examples of conclusive claims from PubMed abstracts.

PMID_Ab^a^	Claim
19322566.ab.15	*CONCLUSION*: A combination of GTI-2040, capecitabine and oxaliplatin is feasible in patients with advanced solid tumors.
28101592.ab.10	In *conclusion*, FCM regimen allows excellent long-lasting response in previously untreated patients with FL.
21198717.ab.10	WHAT IS NEW AND *CONCLUSION*: The use of novel agents such as thalidomide, bortezomib and lenalidomide for RRMM is highly prevalent in France from the first relapse.
23197589.ab.8	We *conclude* that intraventricular rituximab in combination with MTX is feasible and highly active in the treatment of drug-resistant CNS NHL that is refractory or unresponsive to IV rituximab.

^a^PMID_Ab: PubMed reference number, abstract, sentence in which the information appears.

**Table 2:** Examples of SemRep semantic predications based on a biomedical claim.

Example claim			Predicate		Object
**19322566.ab.15 CONCLUSION: A combination of *GTI-2040*, *capecitabine* and *oxaliplatin* is feasible in patients with *advanced solid tumors*.**					
	Advanced Malignant Solid Neoplasm	PROCESS_OF		Patients	
	GTI2040	TREATS		Patients	
	*GTI2040*	*TREATS(INFER)*		*Advanced Malignant Solid Neoplasm*	
	capecitabine	TREATS		Patients	
	*capecitabine*	*TREATS(INFER)*		*Advanced Malignant Solid Neoplasm*	
	oxaliplatin	TREATS		Patients	
	*oxaliplatin*	*TREATS(INFER)*		*Advanced Malignant Solid Neoplasm*	

### Development of an Algorithm for Discovering Knowledge About Combination Drug Therapy

The UMLS-based SemRep underpins biomedical knowledge discovery applications with its broad coverage and high-quality extracted semantic predications. SemRep enables interpretation of 30 semantic predicates [27], such as “PREVENTS,” “TREATS,” and “INHIBITS.”

To develop our algorithm to automatically discover knowledge about combination drug therapies, we focused on 4 semantic predicates closely related to disease treatment: “TREATS,” “INHIBITS,” “PREVENTS,” and “DISRUPTS” (also inferences with “INFER” such as “TREATS(INFER)”). We also adopted the UMLS Semantic Types “Chemicals and Drugs,” “Disease or Syndrome,” and their child types to restrict the subject and object of SemRep output to drug and disease.

Knowledge about combined drug therapy is detected under the hypothesis that (1) two or more semantic predications (S_1_-P-O and S_i_-P-O, i=2, 3...) are extracted from one conclusive claim in the abstract of a given biomedical publication, and (2) they have an identical object (eg, disease) and predicate (eg, treats) but different subjects (eg, drugs). Referring again to the example used in Table 2, the method provided straightforward discovery of the combined medication knowledge “GTI2040+capecitabine+oxaliplatin-TREATS-Advanced Malignant Solid Neoplasm.”

Generally, the algorithm could be expressed by the following formula (Textbox 1):

**Algorithm text.**

**Algorithm:** Drug combination knowledge discovery

**Input:** Semantic predications S_1_-P-O and S_i_-P-O (i=2, 3...) from one conclusive claim in a biomedical abstract

**Output:** Combined drug therapy knowledge S_1_+S_i_-P-O, where all of the following conditions are satisfied:

1. P∈{TREATS”,“INHIBITS”,“PREVENTS”,“DISRUPTS”}
2. S_1_∈Chemicals and Drugs
3. S_i_∈Chemicals and Drugs, i≥2
4. O∈Disease

### Automated Filtering to Focus on Specific Drug and Disease Names

Knowledge about combined drug therapies primarily pertains to specified drugs and diseases; thus, the generic names of these biomedical entities should be filtered out automatically.

### Filtering out Pharmacologic Actions

**Figure 1:**
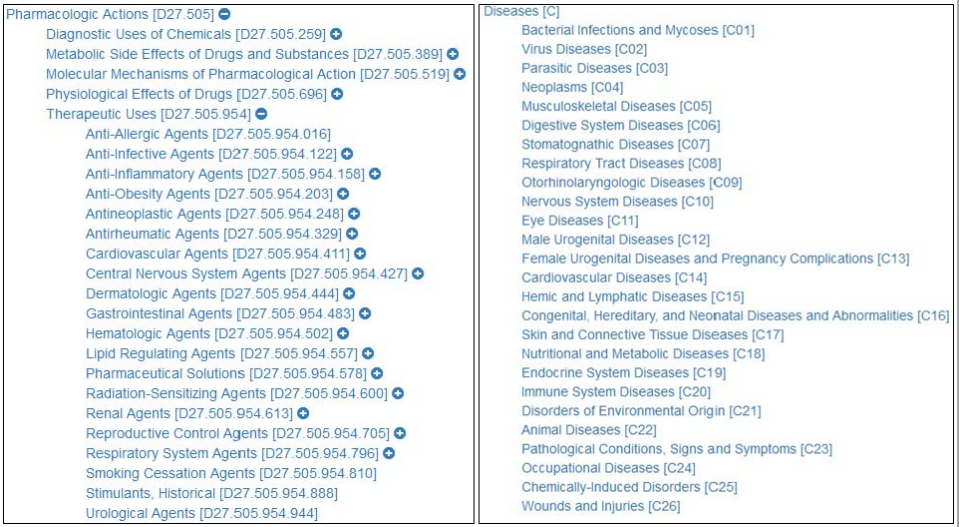
Automatic filtering of pharmacologic actions (left) and generic disease names (right).

In the biomedical domain, the phrase “pharmacologic actions” stands for a broad category of chemical actions and uses that result in the prevention, treatment, cure, or diagnosis of disease. Typical subclasses include “Antineoplastic Agents,” “Lipid Regulating Agents,” and “Anti-Inflammatory Agents”. In the UMLS Metathesaurus, these terms and phrases have been assigned the semantic type “Chemicals and Drugs” and several child types, which would not differ with the specific drug name for our study. To selectively filter out these pharmacologic actions, 497 headings from the MeSH thesaurus were systematically collected based on the tree structure shown in Figure 1 (left).

### Filtering out the Generic Names of Diseases

The top-level names of diseases were automatically filtered by disease (class C in the MeSH tree structure) and its direct hyponyms with tree number from C01 to C26, totaling 27 terms. This filtering was applied because the terms are better regarded as classes of disorders rather than specific diseases (Figure 1 [right]).

### The Construction and Visualization of Knowledge Graph About Combined Drug Therapy

The knowledge graph is an evolving technology widely used for massive knowledge organization and presentation in the era of big data and artificial intelligence due to its ability to mine machine-understandable knowledge and information. In terms of data structure and storage, knowledge graphs store knowledge in the form of subject-predicate-object (usually called a semantic triple). Traditionally, to visualize a domain knowledge graph, the subjects and objects of triples are intuitively displayed as nodes in a graph, with the predicates presented as various edges linked to subjects and objects accordingly.

**Figure 2:**
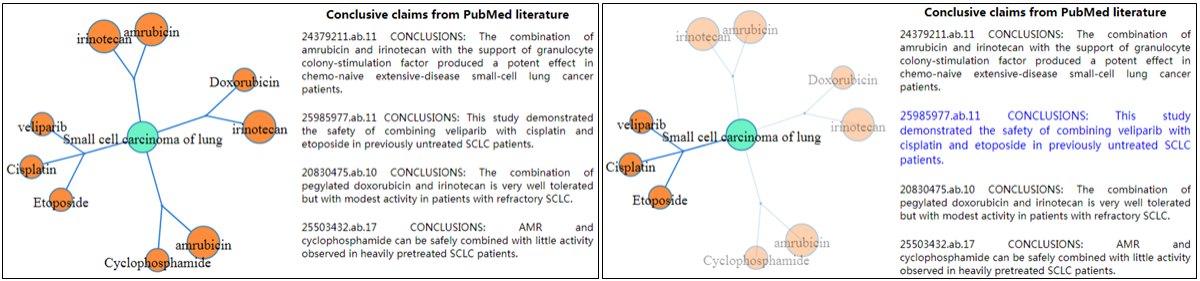
Customized knowledge graph visualization (left) and the conclusive claim being highlighted (right).

In this paper, to emphasize the combined drugs, knowledge about combined drug therapies (S_1_+S_i_)-P-O (i≥2) discovered by the proposed algorithm will be demonstrated such that the combined drugs will be first bound together and then directed to a specified disorder, while the supporting conclusive claims are shown on the right (Figure 2, left). Upon selecting the linked edge of interest, the specific claim regarding the combined medication will be amplified and highlighted (Figure 2, right). The JavaScript libraries Data-Driven Document (D^3^) [28] was utilized to visualize the knowledge graph.

## Results

### Data Acquisition and Experimental Setup

**Figure 3:**
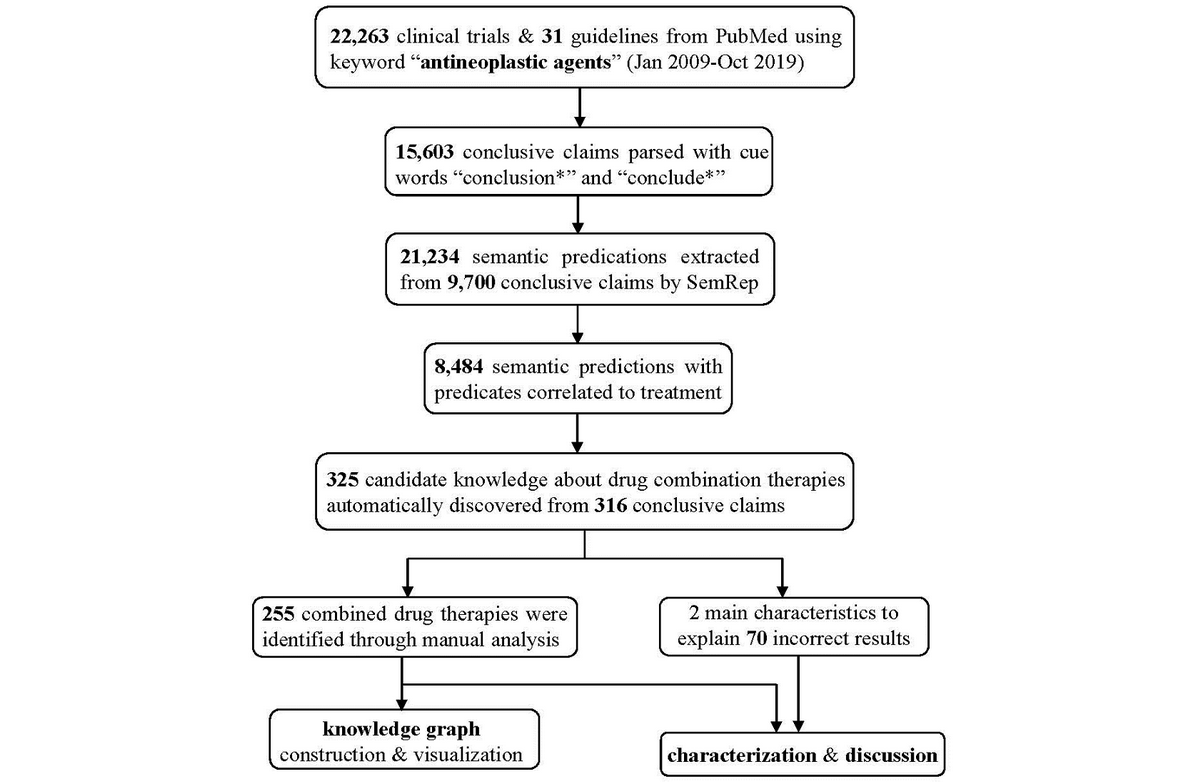
Study design.

A summary of the steps taken to discover and identify combined drug therapies is shown in Figure 3. We retrieved 22,263 clinical trial reports and 31 clinical practice guidelines of PubMed abstracts for algorithm verification and validation, with the subject majored on “antineoplastic agents” for drug restriction (Jan 2009 to Oct 2019). The following PubMed queries were used to identify clinical articles:

1. Clinical trial reports: ((“clinical trial” [Publication Type] OR “clinical trial, phase I” [Publication Type] OR “clinical trial, phase ii” [Publication Type] OR “clinical trial, phase iii” [Publication Type] OR “clinical trial, phase iv” [Publication Type]) OR “clinical study” [Publication Type]).
2. Clinical practice guidelines: “guideline” [Publication Type]

Using the keywords “conclusion*” and “conclude*”, 15,603 conclusive claims were locally segmented and preserved, then pushed into the batch mode of SemRep for semantic predication extraction. Initially, there were 21,234 semantic predications extracted from 9700 conclusive claims, while 8484 predications had semantic predicates focusing on disease treatment (“TREATS,” “INHIBITS,” “PREVENTS,” and “DISRUPTS”). We then employed the automated algorithm to discover knowledge about combined drug therapies while automatically filtering out pharmacologic actions and generic disease names. As a result, 325 candidate groups of semantic predications about combined drug therapies were discovered from 316 conclusive claims for further analysis and characterization.

### Evaluation

Two biocurators annotated 325 candidate groups of semantic predications about combined medications, which were automatically discovered by the algorithm based on SemRep’s semantic predications from 316 conclusive claims. The primary criteria of the biocuration process were that (1) the discovered drugs were combined to treat the specific disease in a given claim, and a single therapy should be identified; (2) the efficacy of combined therapeutic must be promising and negation was disallowed; and (3) the drug name and disease name should be properly recognized by SemRep. Both biocurators independently evaluated all the candidates groups and identified 255 and 239 combined drug therapies (agreement rate 93.73%). Their disagreements mainly lay in the SemRep object “advanced cancer,” which came from more specific terminal malignancies studied in the conclusive claims (such as “advanced carcinomas of the head and neck” in PMID [PubMed ID] 21947123). After consulting a biomedical scientist with specific clinical knowledge, we accepted this kind of text mapping, acknowledging that advanced cancers usually spread from where they started to other parts of the body. Eventually, 255 of 325 (78.46%) groups of semantic predications were identified to be accurate drug combinations (Multimedia Appendix 1), while 70 were determined to be inaccurate and further classified into 2 categories: limitations of SemRep and limitations of proposal.

### Knowledge Graph Construction Based on Identified Knowledge About Combined Medications

**Figure 4:**
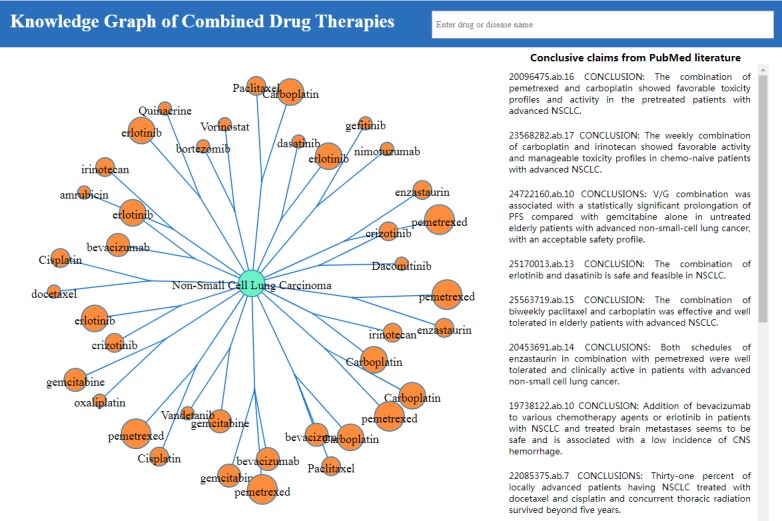
Knowledge graph of combined drug therapies centered at “Non-Small Cell Lung Carcinoma”.

Of the 255 identified combined drug therapies, 210 (82.35%) represented combinations of two drugs, 43 (16.86%) combined 3 agents, and 2 (0.78%) included 4 combined medications. These accurate drug combinations as well as their supporting claims were then used to build the knowledge graph based on customized data structure ((S_1_+S_i_)-P-O, i≥2). Figure 4 shows a snapshot by searching for “Non-Small Cell Lung Carcinoma”.

### Characteristics of Inaccurate Results

There were 70 groups of semantic predications from the automated discovery which, upon manual inspection, were deemed inaccurate due to limitations of SemRep (25/70, 35.7%), or limitations of the proposed algorithm (45/70, 64.3%). These were further categorized to include Named Entity Recognition (NER; 8/70, 11.4%) and Semantic Predicate Extraction (SPR) error (17/70, 24.3%), as well as single therapy (40/70, 57.1%) or multiple combined therapies (5/70, 7.1%). Table 3 summarizes the inaccurate results and their characteristics.

### Limitations of SemRep

NER is one of the key tasks for knowledge discovery and information retrieval, usually implemented before SPR. In SemRep, NER will be executed by MetaMap, a highly configurable program mapping the biomedical entity to the UMLS Metathesaurus. However, due to the relatively limited coverage of the UMLS Metathesarus or the ambiguity of a given biomedical text, MetaMap may inadequately identify an entity, resulting in an improper semantic subject or object. For the first example in Table 3, “ED-SCLC” represents the abbreviation of “extensive-stage disease, small-cell lung cancer,” which is expected to map to “Small cell lung cancer extensive stage” (Concept Unique Identifier: C0278726), but not “Widespread Disease” (CUI: C0849867).

SPR error is another example of SemRep imprecision. In particular, the keyword “failed” was sometimes ignored by SemRep when it appeared in a biomedical text (see the second example in Table 3), resulting in the semantic predicates “TREATS” instead of “NEG_TREATS.” To reduce frequency at which negative predications are extracted, we plan to preprocess conclusive claims to filter out negations before SemRep interpretation.

### Limitations of the Proposed Algorithm

A majority (40/70, 57.1%) of inaccurate results from the automated algorithm were references to single therapies primarily in comparative clinical studies of two or more individual agents. SemRep’s predicate “COMPARED_WITH” may provide a means to filter out these predications. It is common for two or more combined drug therapies to be studied in one published clinical trial (the last claim in Table 3). Future work will focus on these issues to improve the performance of the proposed algorithm.

**Table 3:** Characteristics of inaccurate results from proposed automatic algorithm.

Explanation		No.	Example	PMID_tx^a^
**Limitations of SemRep**				
	NER error	8	bevacizumab-TREATS-*Widespread Disease* Cisplatin-TREATS-*Widespread Disease* Etoposide-TREATS-*Widespread Disease*	19826110.ab.12 CONCLUSION: The addition of bevacizumab to cisplatin and etoposide in patients with *ED-SCLC* results in …
	SPR error	17	ASA 404-*TREATS*-Non-Small Cell Lung Carcinoma Carboplatin-*TREATS*-Non-Small Cell Lung Carcinoma Paclitaxel-*TREATS*-Non-Small Cell Lung Carcinoma	21709202.ab.11 CONCLUSION: The addition of ASA404 to carboplatin and paclitaxel, although generally well tolerated, *failed* to improve frontline efficacy in advanced NSCLC.
**Limitations of proposal**				
	Single Therapy	40	pemetrexed-TREATS-Non-small cell lung cancer metastatic erlotinib-TREATS-Non-small cell lung cancer metastatic	23661337.ab.9 CONCLUSION: Both pemetrexed and erlotinib had *comparable* efficacy in pre-treated patients with metastatic NSCLC.
	Multiple combined therapies	5	Custirsen-TREATS-Hormone refractory prostate cancer docetaxel-TREATS-Hormone refractory prostate cancer Mitoxantrone-TREATS-Hormone refractory prostate cancer	21788353.ab.15 CONCLUSION: Custirsen plus *either* docetaxel *or* mitoxantrone was feasible in patients with progressive mCRPC following first-line docetaxel therapy.

^a^PMID_tx: PubMed identifier, abstract, sentence number, and associated text

## Discussion

### Major Patterns of Combinations According to the Mechanisms of Drugs Being Combined

Among 255 identified combined drug therapies, there were 142 specific drugs after duplicate removal. Classifying by mechanism, 125/142 (88.03%) are antineoplastic agents with 46/142 (32.39%) cytotoxic drugs, 59/142 (41.55%) targeted drugs, 11/142 (7.75%) immunotherapies, 3/142 (2.11%) hormonal drugs, and 6/142 (4.23%) other antineoplastic agents or adjuvant drugs.

We investigated the patterns of identified knowledge based on the mechanism of antineoplastic agents and counted the number of drug combinations under each pattern (Table 4). Although there were fewer cytotoxic drugs than targeted agents, the most common pattern (68/255, 26.67%) were combinations of two cytotoxic drugs, which may provide statistical and practical insights to study new combination of antineoplastic agents for precision medicine. If an antineoplastic agent A produces the same cytotoxic effect as another drug B, and a combination of A and a third cytotoxic agent C has been approved to treat a specific malignancy, our findings suggest the feasibility of a novel combination of B and C (Table 4). Other possible combinations such as A+B and A+B+C may also be valuable to explore. Since various combinations can be followed to develop combined therapies, it is important to be aware of and remain current on all available clinical studies that may be relevant. Our knowledge graph will not only provide a visual representation of existing drug combinations, but also assist practitioners and experts to take full advantage of publicly disseminated clinical trials.

**Table 4:** Major patterns of combined medication based on mechanisms of antineoplastic agents.

Combinations	Number of Instances
Cytotoxic + Cytotoxic	68
Targeted + Cytotoxic	45
Targeted + Targeted	22
Targeted + Cytotoxic + Cytotoxic	17
Cytotoxic + Other antineoplastic agent/adjuvant drugs	15
Immunotherapy + Targeted	13
Targeted + Other antineoplastic agent/adjuvant drugs	11
Immunotherapy + Cytotoxic	10
Cytotoxic + Cytotoxic + Cytotoxic	6
Others	48

### Combined Drug Therapies Discovered in Published Clinical Trials and Clinical Practice Guidelines

All of the combined drug therapies identified in this study were from published clinical trial reports, none of which has been included in clinical practice guidelines. We identified 28 of 31 (90.32%) abstracts in guidelines listed in PubMed by searching “antineoplastic agents” (Jan 2009 to Oct 2019). However, only 4/31 (12.90%) contained conclusive claims with the key words “conclusion*” and “conclude*”, with topics for single therapy (PMID: 20390116), intra-arterial chemotherapy (PMID: 23828325), curriculum in surgical oncology (PMID: 27145931), or drug management (PMID: 30381047). We then manually read the remaining guidelines and identified two combined drug therapies in one publication (PMID:21821491). We thus conclude that our method of parsing conclusive claims from PubMed abstracts may not be suitable for clinical practice guidelines, as a considerable number of these publications (87.10%) do not contain the necessary key words. Using structured abstracts after conversion or applying additional key words like “summar*” may improve the acquisition of conclusive claims. Although mentions of combined drug therapies are limited in clinical practice guidelines, our study focused on the discovery of combination therapies from published clinical trials, which inform the development of clinical practice guidelines.

### The Markers to Identify Potential Combined Drug Therapies

The word “combin*” (namely “combine” or “combination”) is generally used to indicate the combined medication, an assumption affirmed by the data sampled here. Among 316 conclusive claims to automatically identified in this study (Table 5), 171 (54.11%) contain the marker “combin*” and 170 discuss drug combinations, while one described a combination of a drug and radiotherapy. We also noted “coadministration” (2 occurrences) and “co-administered” (1 occurrence) are markers similar to “combin*”, as is “regimen” (22 occurrence, 21 of which were for combined drug therapies) being an abbreviation of “antineoplastic combined chemotherapy regimens” [29]. These markers will become key features in the development of our next deep learning–based knowledge discovery algorithm. After SemRep extraction of semantic relations from conclusive claims in the biomedical literature, we plan to add the Bidirectional Encoder Representations from Transformers [30] model as a binary classifier using annotated data from two dimensions: the supporting conclusive claims and the factuality of semantic predications. The claims containing at least one of the identified markers will be used to classify the corresponding groups of semantic predications into positive knowledge about combined drug therapies.

**Table 5:** Major makers to identify combined drug therapies.

Markers	Occurrence	Combined drug therapy	Other therapy
combin*	171	170	drug & radiotherapy
coadministration	2	2	N/A^a^
co-administered	1	1	N/A
regimen (without markers above)	22	21	Single therapy

^a^N/A: not applicable.

### The Utility and Major Applications of the Knowledge Graph for Combined Drug Therapies

The knowledge graph of combined drug therapies will be an appropriate supplement to most leading knowledge bases, similar to SemMedDB [31], which is a widely used publicly available repository extracted from biomedical literature by SemRep. However, the lack of knowledge concerning combinatorial effects is an important limitation of SemMedDB. Our study seeks to fill this gap by providing the combined medications to enrich the coverage and information provided by SemMedDB and other biomedical knowledge systems.

The proposed knowledge graph has two major applications. An information retrieval system can utilize the knowledge from our graph to integrate various external sources of knowledge and information. Since the subjects and objects of the presented combined medications were drawn from the UMLS Metathesaurus by SemRep, it should be straightforward to integrate our graph with UMLS’s source vocabularies for information retrieval, such as DrugBank, Disease Ontology, NCI thesaurus, SNOMEDCT, etc. Another major application is precision medicine and clinical decision-making support. Combined drug therapies provide an alternative to conventional single therapies especially for malignant disorders. In order to pursue clinical and therapeutic approaches to optimal disease management based on individual variations in a patient's genetic profile, it is useful for an expert working with the treatment of a specific cancer to know which other therapies could also fit in that clinical practice. Manually reading the tremendous literature to find available combinations is undoubtedly laborious and time-consuming. Our knowledge graph will help experts quickly and easily identify efficacious combined therapies that may not be immediately evident by a manual survey of published clinical studies.

### Conclusions

We have shown that semantic predications extracted from large-scale conclusive claims in biomedical research literature can be used to automatically discover and build a customized knowledge graph to represent existing knowledge about combination therapies. We found that additional filtering and evaluation steps were needed to accurately identify drug combinations from candidate results automatically discovered by the proposed algorithm. From 22,263 published clinical trials retrieved from PubMed, we automatically discovered 325 candidate groups of semantic predications, 255 of which (78.46%) were manually verified as accurate. Two major categories and four subcategories were identified to characterize 70 inaccurate results. To address this precision error, we conclude that additional filtering, context analysis, and feature extraction are required to eliminate single therapies and incorrect semantic predications of SemRep output through active learning [32] or a factuality analyzer program [33].

The proposed algorithm can be generalized to automatically discover generic combined medications for all human disorders, not just malignant neoplasms. It is also likely that a larger number of combined drug therapies could be identified in other types of biomedical publications, such as meta-analysis and comparative studies, in which combined medications are frequently addressed.

By characterizing the major patterns of combinations according to the individual drug mechanisms, we found that combinations of two cytotoxic drugs are the most common for cancer treatment. Moreover, four apparent markers (“combin*”, “coadministration”, “co-administered” and “regimen”) were extracted as key features to further develop the machine learning-based knowledge discovery algorithm.

## Appendix

Multimedia Appendix 1 Discovered combined drug therapies.

## Abbreviations

CUI: Concept Unique Identifier
MeSH: Medical Subject Headings
NER: Named Entity Recognition
PMID: PubMed ID/reference number
SemRep: Semantic Knowledge Representation
SPR: Semantic Predicate Extraction
UMLS: Unified Medical Language System
